# Longitudinal trajectories of adiposity-related measures from age 2–5 years in a population of low-income Hispanic children

**DOI:** 10.1038/s41390-020-1099-8

**Published:** 2020-08-04

**Authors:** Catherine M. Crespi, Shuang Gao, Alexandra Payne, Tabashir Z. Nobari, Analissa Avila, Claudia Nau, Shannon E. Whaley, May C. Wang

**Affiliations:** 1 Department of Biostatistics, Fielding School of Public Health, University of California Los Angeles, Los Angeles, CA USA; 2 Penn State College of Medicine, Hershey, PA USA; 3 Department of Public Health, California State University Fullerton, Fullerton, CA USA; 4 Kaiser Permanente Southern California, Pasadena, CA USA; 5 PHFE WIC Program, Irwindale, CA USA; 6 Department of Community Health Sciences, Fielding School of Public Health, University of California Los Angeles, Los Angeles, CA USA

## Abstract

**Background:**

We estimated longitudinal trajectories of body mass index (BMI) *z*-score and percentile, weight for height (WFH) *z*-score and percentile, and percentage of the 95th BMI percentile (BMIp95) among low-income Hispanic children ages 2–5 years to provide normative data for this population and compare the behavior of different measures.

**Methods:**

Longitudinal height and weight measurements obtained from 18,072 Hispanic children aged 2–5 years enrolled in the Special Supplemental Nutrition Program for Women, Infants and Children in Los Angeles County were analyzed. Trajectories of adiposity-related measures were estimated using mixed models, stratified by sex and BMI percentile at age 2 years.

**Results:**

For children in the 5th–85th BMI percentile at age 2 years, all adiposity-related measures rose during ages 2–3.5 years; during ages 3.5–5 years, BMI-based measures increased, BMIp95 decreased, and WFH-based measures were stable. For children exceeding the 85th BMI percentile at age 2 years, measures generally trended downward during ages 2–5 years, except for BMIp95, which had variable trends.

**Conclusions:**

Adiposity measures changed at different rates as children grew during ages 2–3.5 years compared to ages 3.5–5 years, and different measures displayed different trends. Studies should consider examining multiple measures and focusing on change relative to a comparison group.

**Impact:**

- To address the childhood obesity epidemic, information on normative trajectories of adiposity-related measures in at-risk populations of young children is needed.
- Longitudinal analysis of data collected from low-income Hispanic children during ages 2–5 years revealed different patterns for different adiposity measures and for ages 2–3.5 years versus 3.5–5 years.
- Child obesity studies should consider examining multiple adiposity measures and focus on change relative to a comparison group to avoid misinterpreting longitudinal patterns.

## Introduction

Childhood obesity is prevalent in the United States^1^ and worldwide.^2^ In the U.S., obesity prevalence ranged from 14% in ages 2–5 years to 21% in ages 12–19 years in 2015–2016.^1^ Global prevalence of obesity increased from 0.7% to 5.6% in girls in 1975–2016 and from 0.9% to 7.8% in boys aged 5–19 years.^2^ Childhood obesity is associated with increased risk of obesity later in life^3^ and serious chronic health conditions.^4^ As a result, considerable resources have been invested in intervention programs and policies designed to prevent and treat childhood obesity.^5–7^

To evaluate the effectiveness of interventions and monitor trends, it is important to understand normative trajectories of adiposity (body fatness) in children. Direct measures of adiposity such as underwater weighing, air displacement plethysmography, and dual-energy X-ray absorptiometry (DXA) are burdensome and expensive, require specially trained staff, and can involve exposure to radiation (e.g., DXA) and thus are impractical for clinical use.^8^ Instead, weight and height have been used to create indices of adiposity such as body mass index [BMI; weight (kg)/height (m)^2^] and weight for height [WFH, weight (kg)/height (m)], which can be expressed as *z*-scores and percentiles in relation to a reference population. In general, BMI *z*-scores and percentiles have been found to be less informative for severely obese children^9^ and may be poor predictors of adiposity changes over time.^10^ Flegal et al. have suggested that percentage of the 95th percentile of BMI (BMIp95) may have better validity for assessing obesity among the severely obese,^11^ because it provides a better fit to extreme percentiles of BMI than do the percentiles extrapolated from Centers for Disease Control and Prevention (CDC)-supplied growth chart parameters. For children under the age of 5 years, WFH *z*-scores and percentiles have sometimes been used to assess overweight and obesity^12^; however, little is known about their validity.

In the U.S., children from low-income families (of all ethnicities) and ethnic minority groups are at especially high risk for obesity.^13^ The prevalence of obesity among children ages 2–19 years from low-income households is nearly twice that of children from households with incomes >350% of the federal poverty level (20% versus 11%).^13^ The prevalence rates of obesity for Hispanic children and non-Hispanic black children are 26% and 22%, respectively, compared to 14% for non-Hispanic white children.^1^

The objective of this study was to estimate and compare the trajectories of adiposity-related measures calculated from height and weight in a large sample of low-income Hispanic children ages 2–5 years participating in the Special Supplemental Nutrition Program for Women, Infants and Children (WIC) in Los Angeles County, where >60% of infants are enrolled in WIC and >80% of WIC participants are Hispanic (http://lawicdata.org/). Children of Hispanic origin make up an increasing proportion of U.S. youth, with percentages rising from 17% in 2000 to 26% in 2018.^14^ Most Hispanic children in the U.S. live in households with income <200% of the federal poverty level.^15^ However, normative data on longitudinal trajectories of adiposity-related measures for low-income Hispanic children are lacking.

## Methods

### Data source

Data were obtained from the WIC Data Mining Project (http://www.phfewic.org/Projects/DataMining.aspx), a research partnership between Public Health Foundation Enterprises WIC, the largest local agency WIC program in the United States, and First 5 LA (http://www.first5la.org), an independent public agency serving Los Angeles County. The WIC Data Mining Project maintains a database that includes height and weight measurements on all WIC participants residing in Los Angeles County and served by any of the seven local agency WIC programs in the county. WIC is a federal nutrition assistance program that provides supplemental foods, nutrition education, breastfeeding support, and referrals to medical and social services for infants, children aged up to 5 years, and pregnant or postpartum women from low-income households.^12^ Participating children’s height and weight are obtained approximately every 6–12 months by WIC staff using a standardized protocol that calls for removal of shoes and outerwear before measuring children; height is measured to the nearest quarter inch and weight to the nearest quarter pound; further details are provided in an earlier paper.^16^ These measurements have been shown to have high accuracy.^16^

For this analysis, a sample that included children with at least 3 height and weight measurements between ages 2 and 5 years was used. Observations with biologically implausible values according to CDC guidelines (*z*-scores of +8 for weight and BMI and +4 for height) were excluded.^17^ This yielded a sample of 18,072 children. The children were born in 2002–2011, and the data were collected in 2004–2016.

### Demographic variables

Child birth date, sex, age, race/ethnicity, language preference, and household income as reported by the mother or guardian during WIC certification visits were obtained from the WIC database. Household income was categorized with reference to the federal poverty level.

### Anthropometric measures

Our main objective was to examine trajectories of adiposity measures that combine height and weight information. However, we also examined trajectories of height and weight to aid interpretation of results. We calculated the following measures for each height–weight observation: raw height, height for age (HFA) *z*-score, and HFA percentile; raw weight, weight for age (WFA) *z*-score, and WFA percentile; raw weight for height (WFH), WFH *z*-score, and WFH percentile; raw BMI, BMI *z*-score, and BMI percentile; raw triponderal mass index (TMI, defined as kg/m^3^); and percentage of the 95th percentile of BMI (BMIp95). All *z*-scores and percentiles were sex and age adjusted and calculated using the SAS program for the 2000 CDC growth charts.^17^ BMIp95 is calculated by expressing BMI as a percentage of the sex- and age-specific 95th percentile of the 2000 CDC reference population. For example, if a child has a BMI of 16 kg/m^2^ and the value of the sex- and age-specific 95th percentile for BMI is 14 kg/m^2^, BMIp95 = (16/14) × 100% = 114%. TMI *z*-scores or percentiles are not provided by CDC growth charts and thus only raw TMI is presented.

Analyses were restricted to children with Hispanic ethnicity. Children with BMI <5th percentile at first measurement were excluded to focus the analysis on children with normal or higher weight status. Initial adiposity status was defined based on BMI percentile at first measurement, with children classified into four groups: 5th–<85th, 85th–<95th, 95th–<99th, and ≥99th percentile at age 2 years.

### Statistical analyses

Analyses were performed using the R software.^18^ The longitudinal trajectories of each measure were estimated using mixed-effects piecewise linear spline models.^19,20^ A piecewise linear spline model divides the *x*-axis into ≥2 segments and fits a linear model to each segment. The linear segments are constrained to join at the break points (knots). This approach was preferred over polynomial growth curves due to the easier interpretation of linear terms as compared to higher-order polynomial terms and to facilitate tests comparing slopes. In our models, a knot was placed at age 3.5 years, allowing different slopes for ages 2–3.5 and 3.5–5 years. A single knot at 3.5 years was suggested by the adaptive splines automated knot selection procedure^21^ and confirmed by comparing the fit of models with knots at 2.5, 3, 3.5, 4, and 4.5 years using Bayesian Information Criterion (BIC). Models with linear segments were also compared to models with quadratic and cubic segments, and linear segments were also preferred according to BIC values. The models included random intercepts and slopes to account for heterogeneity in growth trajectories among children.

One set of models estimated the mean trajectory for each measure for the overall sample stratified by sex. Another set estimated the mean trajectories stratified by sex and initial adiposity status. For each model, we tested: whether the slope for ages 2–3.5 years equals zero; whether the slope for ages 3.5–5 years equals zero; and whether the slope for ages 2–3.5 years equals the slope for ages 3.5–5 years. A zero slope indicates a flat trajectory during that age interval; a non-zero slope indicates a positive or negative trend with age. Inequality of slopes for different age intervals indicates that the value of the slope changes as children age. Given the large sample and number of tests, *p* < 0.01 (two sided) was considered statistically significant.

## Results

The sample of 18,072 children had a mean age at first measurement of 2.5 years (SD 0.3). Table 1 provides additional sample characteristics. More than 70% of the children were from households with incomes <100% of the federal poverty level. About two-thirds of mothers preferred to communicate in Spanish, and one-third preferred English. Thirty-seven percent (*n* = 7301) had BMI percentile exceeding the 85th at first measurement, with 8.8% (*n* = 1585) exceeding the 99th percentile. The total number of height/weight observations was 58,885 and the totals in each age interval (2–3.5 years, 3.5–5 years), sex, and initial adiposity grouping exceeded 1000 (see Appendix). There were no differences in sex, household income, initial BMI percentile, or mother’s preferred language for children with three or more measurements (included in the analysis) compared to those with fewer measurements (excluded from the analysis).

**Table 1:** Characteristics of the sample at the time of first measurement (*n* = 18,072).

	*n*	Percentage
*Sex*		
Female	8958	49.6%
Male	9114	50.4%
*Household income as percentage of federal poverty level*		
0–50%	3958	21.9%
50.1–100%	9113	50.4%
100.1–130%	2833	15.7%
130.1–185%^a^	2168	12.0%
*BMI percentile at first measurement*		
5th–<85th	11,406	63.1%
85th–<95th	3180	17.6%
95th–<99th	1905	10.5%
≥99th	1581	8.7%
*Preferred language of the mother*		
English	6423	35.5%
Spanish	11,622	64.3%
Other	13	0.1%
Missing	14	0.1%

^a^Includes a small number of children (*n* = 121, 0.7%) with household income >185%.

Tables providing detailed information on slope estimates for all of the anthropometric measures stratified by sex and by initial BMI percentile category are provided in the Appendix. We present figures in the main text.

### Height and weight measures

**Fig. 1:**
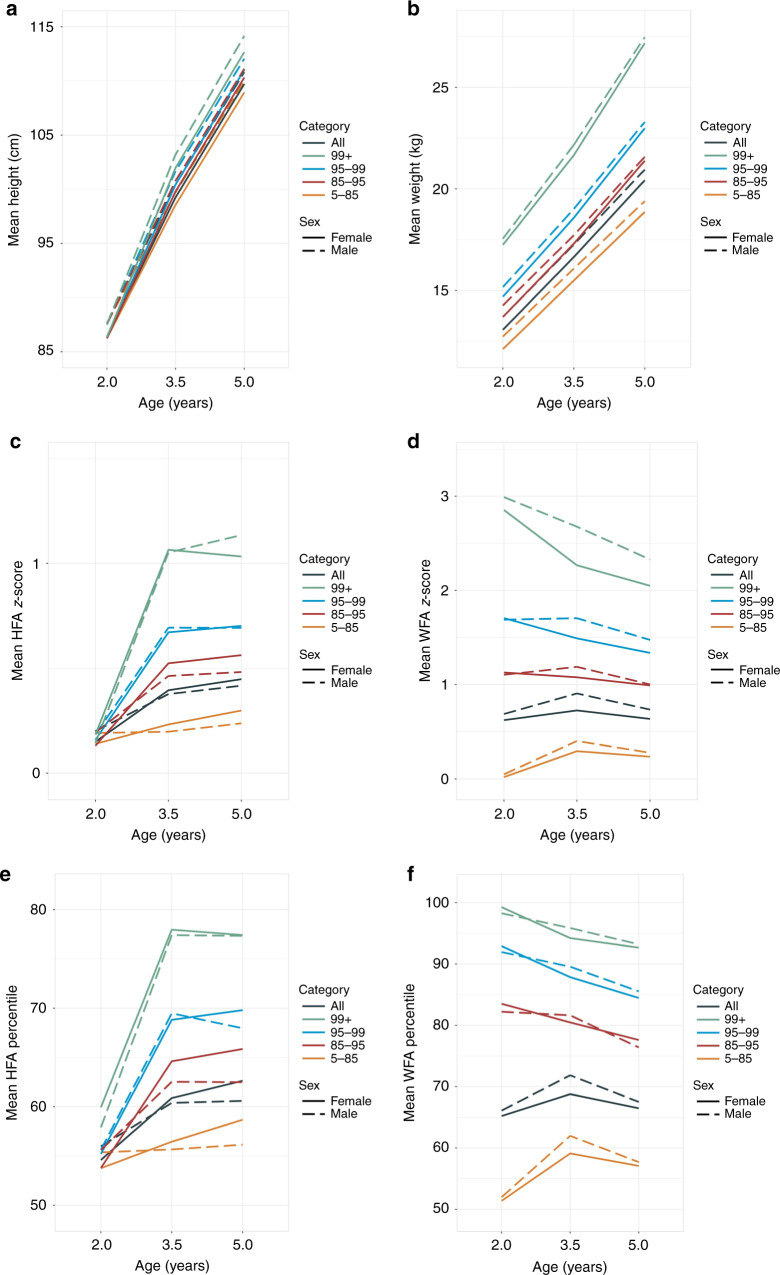
Estimated mean longitudinal trajectories of **a** raw height, **c** height for age (HFA) *z*-score, **e** HFA percentile, **b** raw weight, **d** weight for age (WFA) *z*-score, and **f** WFA percentile, stratified by sex and initial BMI percentile category.

Figure 1 displays mean longitudinal trajectories of raw height, HFA *z*-score, HFA percentile, raw weight, WFA *z*-score, and WFA percentile, stratified by sex and initial BMI percentile. As expected for growing children, raw height and raw weight increased as children grew from ages 2 to 5 years. The rate of height increase slowed after age 3.5 years.

HFA and WFA percentiles and *z*-scores showed similar patterns; we focus on the percentiles. Sex differences were apparent for HFA percentile trends but not for WFA percentile trends; in general, girls showed a more rapid increase in HFA percentile than did boys. There were also different rates of change for ages 2–3.5 years as compared to ages 3.5–5 years. These differences were most apparent for mean HFA percentile of children who were at higher BMI percentiles at age 2 years; these children had rapid increases in HFA percentile until age 3.5 years, after which the trajectory generally flattened. Mean WFA percentile had a quadratic trend for children who were in the 5th–85th BMI percentile at age 2 years and had generally downward slopes for children who began in high BMI categories.

### Raw WFH, BMI, and TMI

**Fig. 2:**
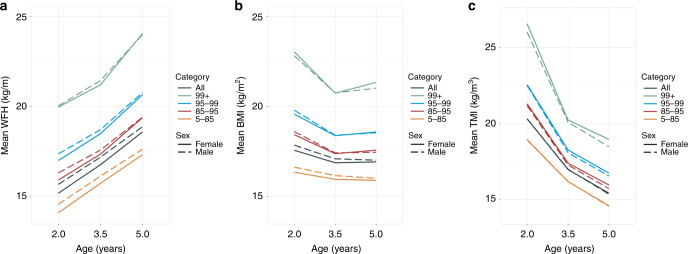
Estimated mean longitudinal trajectories of **a** raw weight for height (WFH), **b** raw body mass index (BMI), and **c** raw triponderal mass index (TMI), stratified by sex and initial BMI percentile category.

Figure 2 shows longitudinal trajectories of raw weight for height (kg/m), body mass index (kg/m^2^), and TMI (kg/m^3^), stratified by sex and initial BMI percentile. Mean raw WFH increased as children grew from ages 2 to 5 years, in all initial BMI percentile categories and both sexes. Mean raw TMI decreased rapidly from ages 2 to 5 years across all groups. Mean raw BMI exhibited significant downward trends for ages 2–3.5 years then became more stable for ages 3.5–5 years.

### WFH and BMI percentiles

**Fig. 3:**
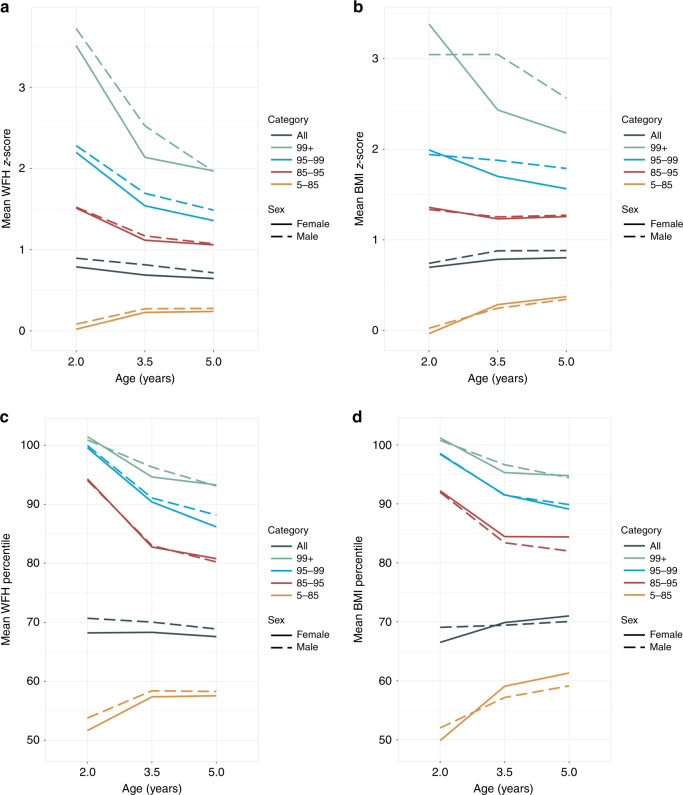
Estimated mean longitudinal trajectories of **a** weight for height (WFH) *z*-score, **b** body mass index (BMI) *z*-score, **c** WFH percentile, and **d** BMI percentile, stratified by sex and initial BMI percentile category.

Figure 3 shows the mean longitudinal trajectories of WFH *z*-scores and percentiles and BMI *z*-scores and percentiles. In the overall sample, the mean WFH percentiles at age 2 years were 68 for girls and 70 for boys; mean BMI percentiles at age 2 years were similar but slightly lower.

For children starting in the 5th–85th BMI percentile, mean BMI percentile increased rapidly during ages 2–3.5 years, then less rapidly through age 5 years. WFH percentile showed similar rapid increases during ages 2–3.5 years, then moderated to flat trajectories. Boys and girls in all of the higher initial BMI percentile categories showed rapidly decreasing mean BMI and WFH percentile during ages 2–3.5 years, then less rapid decreases through age 5 years. During the younger age interval, BMI and WFH percentiles decreased most rapidly among children with initial BMI percentile between the 85th and 95th, followed by the 95th–99th percentile group, then the >99th percentile group.

### BMI and WFH *z*-scores

For both boys and girls in the overall sample, BMI *z*-score showed an upward slope during ages 2–3.5 years, then flat trajectories through age 5 years (Fig. 3). In contrast, WFH *z*-scores showed downward slopes throughout ages 2–5 years for both boys and girls.

For boys and girls with initial BMI percentile of 5th–85th, mean BMI *z*-score increased from age 2 to 5 years but with a slower rate of increase during older ages. Mean WFH *z*-scores increased during ages 2–3.5 years then were flat through age 5 years. For all higher initial BMI percentile categories, mean WFH *z*-score decreased rapidly during ages 2–3.5 years then less rapidly through age 5 years. BMI *z*-scores showed more variable trends. For girls, the mean BMI *z*-score slope was negative during ages 2–3.5 years then became less negative during ages 3.5–5 years. In contrast, for boys, mean BMI *z*-scores were mostly flat or slightly negative as they grew from ages 2 to 5 years, with the exception of rapidly decreasing scores during ages 3.5–5 years for boys initially in the ≥99th BMI percentile.

Some general trends were apparent when comparing longitudinal trajectories of percentiles and *z*-scores for children in the higher initial BMI percentile groups. WFH percentiles decreased most rapidly among the 85th–95th BMI percentile group and least rapidly among the ≥99th percentile group during ages 2–3.5 years. In contrast, WFH *z*-scores decreased most rapidly among children in the ≥99th percentile group and least rapidly among those in the 85th–95th percentile group. BMI percentiles and *z*-scores showed similar patterns for girls but not for boys.

### Percent of the 95th percentile of BMI

**Fig. 4:**
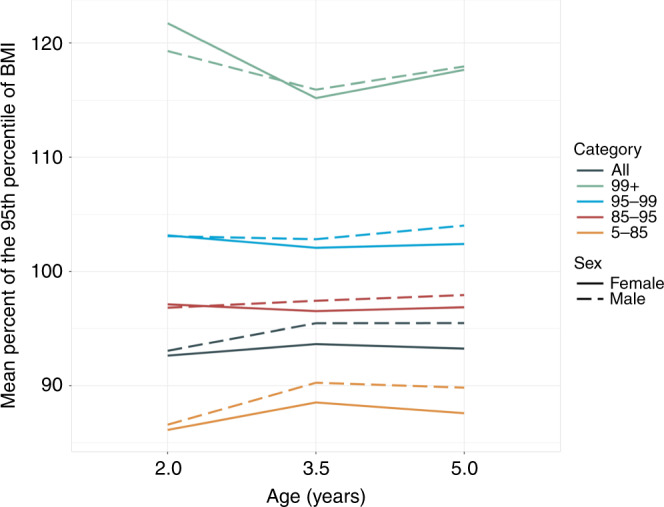
Estimated mean longitudinal trajectories of percentage of the 95th percentile of body mass index (BMIp95), stratified by sex and initial BMI percentile category.

Figure 4 displays longitudinal trajectories of BMIp95. While this measure is most relevant for higher BMI children, we display its trajectory for all initial BMI percentile categories. For boys and girls with initial BMI percentile 5th–85th, mean BMIp95 had a positive slope during ages 2–3.5 years and negative slope during ages 3.5–5 years. Children initially in the 85th–95th BMI percentile showed relatively flat trajectories as they grew from age 2 to 5 years, for both boys and girls. Girls in the 95th–99th percentile category also had a flat trajectory, while that of boys were flat during ages 2–3.5 years, then had rising BMIp95 during ages 3.5–5 years. In the highest initial BMI percentile group (≥99th), mean BMIp95 slopes were negative during ages 2–3.5 years, then positive during ages 3.5–5 years for both boys and girls.

### Variability of slopes

The Appendix tables provide the standard deviation (SD) of the random slope for each measure, which measures the variability of slopes in the population. Trends in the SDs of the random slopes are apparent when examining results by initial status. For BMI and WFH percentile, the SDs decrease as initial adiposity status increases. For BMI and WFH *z*-scores and BMIp95, the SDs increase with increasing initial adiposity status. For BMIp95, children in the highest initial status group had an SD about three times higher than that of the lowest group.

## Discussion

In our study of low-income Hispanic children, our analyses show that the trajectories of different adiposity-related measures from ages 2 to 5 years can be quite different and can depend on sex and initial adiposity status. The rate at which a measure changes as a child grows can also be quite different for younger versus older preschool-aged children.

For children whose BMI was in the 5th–85th percentile at age 2 years, BMI *z*-scores and percentiles, WFH *z*-score and percentiles, and BMIp95 all showed a positive slope as children grew from age 2 to 3.5 years. There are at least two plausible explanations for this pattern. One possibility is that these children were, on average, increasing in adiposity over these 18 months of growth. If true, this implies that ages 2–3.5 years may be a critical window for preventing obesity among children in this population. Another possibility is that these variables increased in value as the children grew without an underlying increase in true adiposity. This could occur if the variables were reflecting changes in weight that are due to increases in height rather than in adiposity. Both explanations could have contributed to the observed pattern.

After age 3.5 years, children who were in the 5th–85th BMI percentile at age 2 years continued on an upward trajectory for BMI percentile and *z*-score while WFH percentile and *z*-score flattened out and BMIp95 took a downward turn. Because our study lacks a gold standard, we are unable to determine which of these measures may reflect the true mean trajectory of adiposity. However, the disparate behavior of these measures raises concern, since on its face it implies three conflicting conclusions: that the children gained adiposity, that they lost adiposity, and that their adiposity status remained stable. This finding suggests that childhood obesity studies whose conclusions are based on examining a single adiposity measure could be misleading. It also highlights the importance of having a comparison group when conducting evaluations of childhood obesity interventions; we may not know which measure is a more accurate indicator of adiposity, but with a comparison group, we can at least make relative comparisons of slopes.

Other longitudinal studies reporting trajectories of adiposity measures among U.S. Hispanic children include that of Min et al.,^22^ who modeled growth curves of raw BMI by ethnicity using data from the Early Childhood Longitudinal Study-Birth Cohort,^23^ a U.S. nationally representative sample of children born in 2001. Although these authors used fractional polynomial models and therefore did not present values for slopes, mean BMI trajectories appeared to slope predominantly downward from ages 2 to 4 years for all ethnic groups assessed (White, African American, Asian, and Hispanic) and for both boys and girls, which is roughly consistent with our results for raw BMI. In this cohort, Hispanic boys and girls had the highest prevalence of overweight and obesity at the age 2 years and preschool (approximately age 4 years) waves, as well as the highest mean BMI over the 2–5-year age interval. Hispanic boys continued to have the highest mean BMI through age 16 years while mean BMI for Hispanic girls remained highest through age 6 years, when African American girls crossed over and exceeded that of Hispanic girls.

In our sample of low-income Hispanic children, the slopes of BMI and WFH *z*-scores and percentiles depended strongly on age, with different slopes for ages 2–3.5 years compared to 3.5–5 years for the majority of our sex and initial status strata. This finding has implications for designing studies and analyzing and interpreting data. In particular, studies including both younger and older children in the 2–5-year age range should anticipate that these groups will have different trajectories. Possible analytic approaches to address these differences include stratifying by age or allowing slopes to vary with age by using flexible models such as polynomials or piecewise linear splines, as we used here.

Among children in high BMI percentiles at age 2 years, mean WFH and BMI *z*-scores and percentiles mostly trended downward through age 5 years, with more rapid declines at younger ages. This is consistent with the findings of Freedman et al.,^24^ who analyzed data from the CDC’s Pediatric and Nutrition Surveillance Study of mostly low-income children and reported declines in BMI *z*-score among children who were severely obese at first examination (mean age of about 3 years). Some of the decline among high-adiposity groups might be attributable to regression to the mean, whereby unusually large or small measurements tend to be followed by measurements that are closer to the mean.^25^ However, there were some clear sex differences. Most notably, among children exceeding the 99th BMI percentile at age 2 years, mean BMI *z*-scores for girls declined rapidly through age 3.5 years, whereas scores for boys remained stable (Fig. 3b). BMIp95 also showed more downward movement for girls as compared to boys for this group (Fig. 4).

BMIp95 has been recommended as superior for tracking and monitoring severely obese children.^9,11,26^ In our sample of low-income Hispanic children, mean BMIp95 was relatively flat during ages 2–5 years among children starting at the 85th–95th and 95th–99th BMI percentiles. If these children were, on average, maintaining their true relative adiposity status during this period, this would support BMIp95 as a reliable indicator for tracking purposes for such children. However, BMIp95 trends were erratic for children whose BMI percentile was ≥99th at age 2 years, with a downward slope during ages 2–3.5 years and upward slope during ages 3.5–5 years. This raises questions about interpreting BMIp95 changes in the most severely obese young children over long periods of time. The SD of the random slopes for BMIp95 was also highest for the most severely obese group, indicating that these children had highly variable trends.

The 2000 CDC reference growth charts are based on nationally representative samples from surveys conducted from 1963 to 1994,^27^ when the percentage of the U.S. population that is Hispanic was much lower than current population levels^28^ and the prevalence of overweight and obesity among children was also lower.^29,30^ In our overall sample, sex- and age-adjusted mean weight for height and BMI all exceeded the median CDC reference values throughout the 2–5-year age interval. This is consistent with the reported higher prevalence of overweight and obesity among Hispanic children.^1^ In the Early Childhood Longitudinal Study-Birth Cohort, mean trajectories of raw BMI for all sex and ethnic groups exceeded the CDC and World Health Organization reference medians, with Hispanic boys and girls the highest from ages 2 to 5 years.^22^

Results stratified by BMI status at age 2 years indicated that children initially at high BMI percentiles gained height at a very rapid rate during the next 12–18 months while their WFA trajectories decreased. This suggests that many children in this group “grew into” their weight. This is consistent with their downward trends in WFH and BMI *z*-scores and percentiles.

Strengths of this study include the large sample size and number of longitudinal measurements. The height and weight measurements were taken by trained professionals using a standard protocol and have demonstrated accuracy.^16^ However, our sample may not be fully representative of the U.S. population of low-income Hispanic children in this age range. All children were Los Angeles County residents and enrolled in the WIC program. Our data did not include direct measures of adiposity such as hydrostatic underwater weighing, DXA, or magnetic resonance imaging and thus we could not address the validity of the various adiposity measure.

## Conclusion

There is an urgent need to understand measures of adiposity in children so that we can properly interpret secular trends, identify populations in need of intervention, evaluate interventions, and make effective policy decisions. Our results indicate that BMI and WFH *z*-scores and percentiles and BMIp95 have very different trajectories in low-income preschool-aged Hispanic children and that these indicators can change at different rates during ages 2–5 years. More research is needed to assess the validity of various anthropometric indices of adiposity in young children and understand how their use may bias estimates of associations.

## Supplementary information

Appendix
