# Supplementary material for: Longitudinal trajectories of adiposity-related measures from age 2–5 years in a population of low-income Hispanic children
Source: Pediatr Res. 2020 Aug 4;89(6):1557–64. doi: 10.1038/s41390-020-1099-8 (PMC8163600; doi:10.1038/s41390-020-1099-8)
Supplement: Supplementary file 1 — Appendix [file 41390_2020_1099_MOESM1_ESM.docx]

**Appendix Table 1. Number of height/weight observations in analyses, stratified by age, sex and initial BMI percentile.**

| **Age** | **Sex** | **Initial BMI percentile** | **n** | **Percent** |
| --- | --- | --- | --- | --- |
| 2.0 - 3.5 years | Female | 5th to <85th | 8,373 | 14.2% |
|  |  | 85th to <95th | 2,209 | 3.8% |
|  |  | 95th to <99th | 1,276 | 2.2% |
|  |  | ≥99th | 1,090 | 1.9% |
|  | Male | 5th to <85th | 8,209 | 13.9% |
|  |  | 85th to <95th | 2,407 | 4.1% |
|  |  | 95th to <99th | 1,462 | 2.5% |
|  |  | ≥99th | 1,078 | 1.8% |
| 3.5 - 5.0 years | Female | 5th to <85th | 10,443 | 17.7% |
|  |  | 85th to <95th | 2,751 | 4.7% |
|  |  | 95th to <99th | 1,637 | 2.8% |
|  |  | ≥99th | 1,416 | 2.4% |
|  | Male | 5th to <85th | 10,250 | 17.4% |
|  |  | 85th to <95th | 3,046 | 5.2% |
|  |  | 95th to <99th | 1,798 | 3.1% |
|  |  | ≥99th | 1,440 | 2.4% |
| Total |  |  | 58,885 | 100% |

**Appendix Table 2. Males: Estimated longitudinal slopes of measures, stratified by initial BMI percentile group. Slopes are estimated mean change per year in the measure.**

| **BMI percentile at first measurement** | **Age 2 - 3.5 years** | | | **Age 3.5 - 5 years** | | |  |  |  | **SD of random slope** |
| --- | --- | --- | --- | --- | --- | --- | --- | --- | --- | --- |
|  | **Slope** | **SE** | **P-value** | **Slope** | **SE** | **P-value** | **Slope diff** | **SE** | **P-value** |  |
| **Height** | | | | | | | | | | |
| 5th to <85th | 8.09 | 0.03 | *** | 6.85 | 0.03 | *** | -1.24 | 0.05 | *** | 0.85 |
| 85th to <95th | 8.84 | 0.06 | *** | 6.87 | 0.06 | *** | -1.96 | 0.10 | *** | 0.79 |
| 95th to <99th | 9.44 | 0.09 | *** | 6.92 | 0.08 | *** | -2.52 | 0.14 | *** | 0.95 |
| ≥99th | 10.45 | 0.14 | *** | 7.29 | 0.12 | *** | -3.16 | 0.21 | *** | 1.41 |
| All | 8.55 | 0.03 | *** | 6.91 | 0.03 | *** | -1.63 | 0.05 | *** | 0.98 |
| **HFA z-score** | | | | | | | | | | |
| 5th to <85th | 0.004 | 0.008 |  | 0.03 | 0.008 | *** | 0.02 | 0.01 |  | 0.19 |
| 85th to <95th | 0.18 | 0.02 | *** | 0.01 | 0.01 |  | -0.17 | 0.03 | *** | 0.17 |
| 95th to <99th | 0.34 | 0.02 | *** | -0.0005 | 0.02 |  | -0.34 | 0.03 | *** | 0.23 |
| ≥99th | 0.61 | 0.04 | *** | 0.06 | 0.03 |  | -0.55 | 0.06 | *** | 0.38 |
| All | 0.12 | 0.007 | *** | 0.03 | 0.007 | *** | -0.09 | 0.01 | *** | 0.22 |
| **HFA percentile** | | | | | | | | | | |
| 5th to <85th | 0.20 | 0.23 |  | 0.32 | 0.23 |  | 0.12 | 0.40 |  | 5.08 |
| 85th to <95th | 4.62 | 0.43 | *** | -0.03 | 0.43 |  | -4.65 | 0.74 | *** | 4.86 |
| 95th to <99th | 9.23 | 0.57 | *** | -1.01 | 0.55 |  | -10.24 | 0.94 | *** | 5.98 |
| ≥99th | 13.00 | 0.74 | *** | -0.04 | 0.65 |  | -13.05 | 1.16 | *** | 7.59 |
| All | 2.94 | 0.19 | *** | 0.14 | 0.18 |  | -2.80 | 0.32 | *** | 5.55 |
| **Weight** | | | | | | | | | | |
| 5th to <85th | 2.23 | 0.02 | *** | 2.21 | 0.02 | *** | -0.02 | 0.02 |  | 0.74 |
| 85th to <95th | 2.30 | 0.04 | *** | 2.57 | 0.04 | *** | 0.27 | 0.05 | *** | 1.00 |
| 95th to <99th | 2.56 | 0.06 | *** | 2.85 | 0.06 | *** | 0.29 | 0.08 | *** | 1.25 |
| ≥99th | 3.08 | 0.10 | *** | 3.53 | 0.09 | *** | 0.45 | 0.13 | *** | 1.79 |
| All | 2.37 | 0.02 | *** | 2.45 | 0.02 | *** | 0.08 | 0.02 | *** | 1.04 |
| **WFA z-score** | | | | | | | | | | |
| 5th to <85th | 0.23 | 0.007 | *** | -0.08 | 0.007 | *** | -0.32 | 0.01 | *** | 0.23 |
| 85th to <95th | 0.06 | 0.01 | *** | -0.12 | 0.01 | *** | -0.18 | 0.02 | *** | 0.26 |
| 95th to <99th | 0.01 | 0.02 |  | -0.15 | 0.02 | *** | -0.16 | 0.03 | *** | 0.28 |
| ≥99th | -0.21 | 0.03 | *** | -0.23 | 0.02 | *** | -0.02 | 0.04 |  | 0.36 |
| All | 0.15 | 0.006 | *** | -0.11 | 0.006 | *** | -0.26 | 0.009 | *** | 0.27 |
| **WFA percentile** | | | | | | | | | | |
| 5th to <85th | 6.68 | 0.21 | *** | -2.86 | 0.20 | *** | -9.54 | 0.34 | *** | 6.20 |
| 85th to <95th | -0.39 | 0.31 |  | -3.49 | 0.30 | *** | -3.09 | 0.49 | *** | 5.39 |
| 95th to <99th | -1.59 | 0.31 | *** | -2.69 | 0.30 | *** | -1.10 | 0.48 |  | 4.45 |
| ≥99th | -1.60 | 0.28 | *** | -1.77 | 0.25 | *** | -0.17 | 0.38 |  | 4.40 |
| All | 3.83 | 0.15 | *** | -2.89 | 0.14 | *** | -6.73 | 0.24 | *** | 6.00 |
| **WFH** | | | | | | | | | | |
| 5th to <85th | 1.04 | 0.01 | *** | 1.00 | 0.01 | *** | -0.05 | 0.02 |  | 0.56 |
| 85th to <95th | 0.85 | 0.03 | *** | 1.22 | 0.03 | *** | 0.37 | 0.05 | *** | 0.77 |
| 95th to <99th | 0.90 | 0.05 | *** | 1.38 | 0.05 | *** | 0.48 | 0.07 | *** | 0.98 |
| ≥99th | 0.94 | 0.09 | *** | 1.70 | 0.08 | *** | 0.76 | 0.13 | *** | 1.44 |
| All | 1.01 | 0.01 | *** | 1.13 | 0.01 | *** | 0.12 | 0.02 | *** | 0.78 |
| **BMI** | | | | | | | | | | |
| 5th to <85th | -0.31 | 0.01 | *** | -0.10 | 0.01 | *** | 0.22 | 0.02 | *** | 0.44 |
| 85th to <95th | -0.79 | 0.03 | *** | 0.02 | 0.03 |  | 0.81 | 0.05 | *** | 0.65 |
| 95th to <99th | -0.93 | 0.05 | *** | 0.101 | 0.05 |  | 1.03 | 0.07 | *** | 0.84 |
| ≥99th | -1.38 | 0.10 | *** | 0.18 | 0.09 |  | 1.56 | 0.15 | *** | 1.30 |
| All | -0.51 | 0.02 | *** | -0.05 | 0.01 | *** | 0.46 | 0.02 | *** | 0.64 |
| **TMI** | | | | | | | | | | |
| 5th to <85th | -1.83 | 0.02 | *** | -1.09 | 0.02 | *** | 0.74 | 0.03 | *** | 0.40 |
| 85th to <95th | -2.61 | 0.04 | *** | -1.03 | 0.04 | *** | 1.58 | 0.06 | *** | 0.51 |
| 95th to <99th | -2.96 | 0.05 | *** | -1.02 | 0.06 | *** | 1.93 | 0.09 | *** | 0.68 |
| ≥99th | -3.96 | 0.13 | *** | -1.08 | 0.12 | *** | 2.89 | 0.20 | *** | 1.46 |
| All | -2.22 | 0.02 | *** | -1.10 | 0.02 | *** | 1.12 | 0.03 | *** | 0.67 |
| **WFH z-score** | | | | | | | | | | |
| 5th to <85th | 0.13 | 0.01 | *** | 0.003 | 0.01 |  | -0.12 | 0.02 | *** | 0.23 |
| 85th to <95th | -0.24 | 0.02 | *** | -0.06 | 0.02 | *** | 0.17 | 0.03 | *** | 0.24 |
| 95th to <99th | -0.39 | 0.02 | *** | -0.14 | 0.02 | *** | 0.25 | 0.04 | *** | 0.25 |
| ≥99th | -0.80 | 0.03 | *** | -0.37 | 0.03 | *** | 0.43 | 0.05 | *** | 0.29 |
| All | -0.05 | 0.008 | *** | -0.07 | 0.008 | *** | -0.01 | 0.01 |  | 0.28 |
| **WFH percentile** | | | | | | | | | | |
| 5th to <85th | 3.08 | 0.30 | *** | -0.08 | 0.30 |  | -3.16 | 0.51 | *** | 6.48 |
| 85th to <95th | -7.37 | 0.36 | *** | -1.86 | 0.37 | *** | 5.52 | 0.60 | *** | 5.84 |
| 95th to <99th | -5.93 | 0.34 | *** | -1.93 | 0.35 | *** | 3.99 | 0.56 | *** | 4.89 |
| ≥99th | -3.06 | 0.32 | *** | -2.15 | 0.31 | *** | 0.91 | 0.48 |  | 4.49 |
| All | -0.44 | 0.22 |  | -0.77 | 0.21 | *** | -0.34 | 0.36 |  | 6.22 |
| **BMI z-score** | | | | | | | | | | |
| 5th to <85th | 0.15 | 0.01 | *** | 0.07 | 0.01 | *** | -0.08 | 0.02 | *** | 0.26 |
| 85th to <95th | -0.05 | 0.02 | ** | 0.01 | 0.02 |  | 0.07 | 0.03 |  | 0.35 |
| 95th to <99th | -0.04 | 0.02 |  | -0.06 | 0.03 |  | -0.02 | 0.04 |  | 0.37 |
| ≥99th | 0.001 | 0.04 |  | -0.32 | 0.04 | *** | -0.32 | 0.06 | *** | 0.40 |
| All | 0.09 | 0.01 | *** | 0.002 | 0.009 |  | -0.09 | 0.02 | *** | 0.29 |
| **BMI percentile** | | | | | | | | | | |
| 5th to <85th | 3.44 | 0.33 | *** | 1.32 | 0.33 | *** | -2.12 | 0.57 | *** | 6.35 |
| 85th to <95th | -5.72 | 0.39 | *** | -0.93 | 0.41 |  | 4.78 | 0.66 | *** | 6.51 |
| 95th to <99th | -4.72 | 0.39 | *** | -1.06 | 0.40 | ** | 3.66 | 0.64 | *** | 5.08 |
| ≥99th | -2.75 | 0.35 | *** | -1.49 | 0.34 | *** | 1.26 | 0.53 |  | 4.91 |
| All | 0.24 | 0.24 |  | 0.43 | 0.23 |  | 0.19 | 0.41 |  | 6.09 |
| **BMIp95** | | | | | | | | | | |
| 5th to <85th | 2.45 | 0.08 | *** | -0.29 | 0.08 | *** | -2.74 | 0.13 | *** | 2.45 |
| 85th to <95th | 0.40 | 0.16 |  | 0.33 | 0.17 |  | -0.07 | 0.25 |  | 3.68 |
| 95th to <99th | -0.18 | 0.26 |  | 0.80 | 0.26 | ** | 0.97 | 0.38 |  | 4.74 |
| ≥99th | -2.24 | 0.54 | *** | 1.35 | 0.50 | ** | 3.60 | 0.81 | *** | 7.30 |
| All | 1.60 | 0.08 | *** | 0.003 | 0.08 |  | -1.60 | 0.13 | *** | 3.59 |

** indicates 0.001<p<0.01; *** indicates p<0.001. Only p-values <0.01 are indicated. P-values are obtained from mixed models in which age is modeled using piecewise linear splines. SD, standard deviation.

**Appendix Table 3. Females: Estimated longitudinal slopes of measures, stratified by initial BMI percentile group. Slopes are estimated mean change per year in the measure.**

| **BMI percentile at first measurement** | **Age 2 - 3.5 years** | | | **Age 3.5 - 5 years** | | |  |  |  | **SD of random slope** |
| --- | --- | --- | --- | --- | --- | --- | --- | --- | --- | --- |
|  | **Slope** | **SE** | **P-value** | **Slope** | **SE** | **P-value** | **Slope diff** | **SE** | **P-value** |  |
| **Height** | | | | | | | | | | |
| 5th to <85th | 8.21 | 0.03 | *** | 6.91 | 0.03 | *** | -1.30 | 0.05 | *** | 0.86 |
| 85th to <95th | 9.05 | 0.07 | *** | 7.00 | 0.06 | *** | -2.05 | 0.11 | *** | 0.84 |
| 95th to <99th | 9.37 | 0.10 | *** | 7.06 | 0.09 | *** | -2.31 | 0.16 | *** | 0.99 |
| ≥99th | 10.43 | 0.13 | *** | 7.12 | 0.13 | *** | -3.31 | 0.21 | *** | 1.46 |
| All | 8.64 | 0.03 | *** | 6.97 | 0.03 | *** | -1.67 | 0.05 | *** | 0.99 |
| **HFA z-score** | | | | | | | | | | |
| 5th to <85th | 0.06 | 0.008 | *** | 0.04 | 0.008 | *** | -0.02 | 0.01 |  | 0.19 |
| 85th to <95th | 0.26 | 0.02 | *** | 0.03 | 0.02 |  | -0.24 | 0.03 | *** | 0.18 |
| 95th to <99th | 0.34 | 0.02 | *** | 0.02 | 0.02 |  | -0.32 | 0.04 | *** | 0.24 |
| ≥99th | 0.59 | 0.03 | *** | -0.02 | 0.03 |  | -0.61 | 0.05 | *** | 0.38 |
| All | 0.16 | 0.007 | *** | 0.04 | 0.007 | *** | -0.13 | 0.01 | *** | 0.22 |
| **HFA percentile** | | | | | | | | | | |
| 5th to <85th | 1.79 | 0.23 | *** | 1.49 | 0.23 | *** | -0.30 | 0.39 |  | 5.29 |
| 85th to <95th | 7.21 | 0.43 | *** | 0.83 | 0.42 |  | -6.38 | 0.73 | *** | 4.93 |
| 95th to <99th | 9.08 | 0.60 | *** | 0.65 | 0.56 |  | -8.43 | 0.97 | *** | 6.53 |
| ≥99th | 12.01 | 0.70 | *** | -0.36 | 0.65 |  | -12.37 | 1.11 | *** | 7.74 |
| All | 4.18 | 0.19 | *** | 1.18 | 0.18 | *** | -3.01 | 0.32 | *** | 5.66 |
| **Weight** | | | | | | | | | | |
| 5th to <85th | 2.25 | 0.02 | *** | 2.25 | 0.02 | *** | -0.0005 | 0.02 |  | 0.74 |
| 85th to <95th | 2.41 | 0.04 | *** | 2.72 | 0.04 | *** | 0.312 | 0.05 | *** | 1.04 |
| 95th to <99th | 2.59 | 0.07 | *** | 2.93 | 0.07 | *** | 0.35 | 0.09 | *** | 1.30 |
| ≥99th | 2.94 | 0.10 | *** | 3.67 | 0.09 | *** | 0.73 | 0.13 | *** | 1.71 |
| All | 2.39 | 0.02 | *** | 2.52 | 0.02 | *** | 0.13 | 0.02 | *** | 1.04 |
| **WFA z-score** | | | | | | | | | | |
| 5th to <85th | 0.18 | 0.007 | *** | -0.04 | 0.007 | *** | -0.22 | 0.01 | *** | 0.23 |
| 85th to <95th | -0.03 | 0.01 |  | -0.06 | 0.01 | *** | -0.02 | 0.02 |  | 0.24 |
| 95th to <99th | -0.14 | 0.02 | *** | -0.10 | 0.02 | *** | 0.04 | 0.03 |  | 0.274 |
| ≥99th | -0.39 | 0.02 | *** | -0.15 | 0.02 | *** | 0.25 | 0.04 | *** | 0.27 |
| All | 0.07 | 0.006 | *** | -0.06 | 0.006 | *** | -0.13 | 0.009 | *** | 0.26 |
| **WFA percentile** | | | | | | | | | | |
| 5th to <85th | 5.16 | 0.21 | *** | -1.35 | 0.198 | *** | -6.51 | 0.32 | *** | 6.81 |
| 85th to <95th | -2.02 | 0.32 | *** | -1.92 | 0.31 | *** | 0.11 | 0.52 |  | 5.16 |
| 95th to <99th | -3.37 | 0.40 | *** | -2.27 | 0.38 | *** | 1.10 | 0.61 |  | 5.51 |
| ≥99th | -3.36 | 0.33 | *** | -1.03 | 0.34 | ** | 2.33 | 0.52 | *** | 4.66 |
| All | 2.38 | 0.16 | *** | -1.53 | 0.15 | *** | -3.91 | 0.24 | *** | 6.56 |
| **WFH** | | | | | | | | | | |
| 5th to <85th | 1.09 | 0.01 | *** | 1.06 | 0.01 | *** | -0.03 | 0.02 |  | 0.57 |
| 85th to <95th | 0.96 | 0.03 | *** | 1.35 | 0.03 | *** | 0.39 | 0.05 | *** | 0.81 |
| 95th to <99th | 0.99 | 0.06 | *** | 1.45 | 0.06 | *** | 0.46 | 0.08 | *** | 1.04 |
| ≥99th | 0.83 | 0.09 | *** | 1.91 | 0.09 | *** | 1.08 | 0.13 | *** | 1.40 |
| All | 1.06 | 0.02 | *** | 1.22 | 0.01 | *** | 0.16 | 0.02 | *** | 0.79 |
| **BMI** | | | | | | | | | | |
| 5th to <85th | -0.27 | 0.01 | *** | -0.04 | 0.01 |  | 0.23 | 0.02 | *** | 0.47 |
| 85th to <95th | -0.70 | 0.03 | *** | 0.13 | 0.03 | *** | 0.84 | 0.05 | *** | 0.69 |
| 95th to <99th | -0.80 | 0.05 | *** | 0.14 | 0.05 |  | 0.94 | 0.08 | *** | 0.90 |
| ≥99th | -1.54 | 0.10 | *** | 0.40 | 0.10 | *** | 1.93 | 0.16 | *** | 1.34 |
| All | -0.47 | 0.02 | *** | 0.03 | 0.02 |  | 0.50 | 0.03 | *** | 0.67 |
| **TMI** | | | | | | | | | | |
| 5th to <85th | -1.83 | 0.02 | *** | -1.06 | 0.02 | *** | 0.77 | 0.03 | *** | 0.42 |
| 85th to <95th | -2.60 | 0.04 | *** | -0.96 | 0.04 | *** | 1.64 | 0.07 | *** | 0.54 |
| 95th to <99th | -2.87 | 0.06 | *** | -1.01 | 0.06 | *** | 1.86 | 0.10 | *** | 0.73 |
| ≥99th | -4.23 | 0.14 | *** | -0.85 | 0.13 | *** | 3.38 | 0.22 | *** | 1.65 |
| All | -2.22 | 0.02 | *** | -1.04 | 0.02 | *** | 1.19 | 0.03 | *** | 0.73 |
| **WFH z-score** | | | | | | | | | | |
| 5th to <85th | 0.14 | 0.009 | *** | 0.008 | 0.009 |  | -0.13 | 0.02 | *** | 0.23 |
| 85th to <95th | -0.26 | 0.02 | *** | -0.04 | 0.02 |  | 0.23 | 0.03 | *** | 0.22 |
| 95th to <99th | -0.44 | 0.02 | *** | -0.12 | 0.02 | *** | 0.32 | 0.03 | *** | 0.24 |
| ≥99th | -0.92 | 0.03 | *** | -0.11 | 0.03 | *** | 0.81 | 0.04 | *** | 0.26 |
| All | -0.07 | 0.008 | *** | -0.03 | 0.008 | *** | 0.04 | 0.01 | ** | 0.28 |
| **WFH percentile** | | | | | | | | | | |
| 5th to <85th | 3.81 | 0.29 | *** | 0.13 | 0.29 |  | -3.68 | 0.49 | *** | 7.30 |
| 85th to <95th | -7.69 | 0.37 | *** | -1.32 | 0.38 | *** | 6.37 | 0.62 | *** | 5.67 |
| 95th to <99th | -6.14 | 0.41 | *** | -2.81 | 0.42 | *** | 3.33 | 0.67 | *** | 5.34 |
| ≥99th | -4.57 | 0.36 | *** | -0.89 | 0.36 |  | 3.67 | 0.57 | *** | 4.87 |
| All | 0.07 | 0.22 |  | -0.49 | 0.21 |  | -0.56 | 0.36 |  | 6.96 |
| **BMI z-score** | | | | | | | | | | |
| 5th to <85th | 0.21 | 0.01 | *** | 0.06 | 0.01 | *** | -0.16 | 0.02 | *** | 0.24 |
| 85th to <95th | -0.09 | 0.02 | *** | 0.02 | 0.02 |  | 0.10 | 0.03 | *** | 0.26 |
| 95th to <99th | -0.19 | 0.02 | *** | -0.09 | 0.02 | *** | 0.10 | 0.04 | ** | 0.28 |
| ≥99th | -0.63 | 0.03 | *** | -0.17 | 0.03 | *** | 0.46 | 0.05 | *** | 0.30 |
| All | 0.06 | 0.009 | *** | 0.01 | 0.008 |  | -0.05 | 0.01 | ** | 0.28 |
| **BMI percentile** | | | | | | | | | | |
| 5th to <85th | 6.14 | 0.31 | *** | 1.50 | 0.31 | *** | -4.64 | 0.52 | *** | 7.05 |
| 85th to <95th | -5.18 | 0.38 | *** | -0.04 | 0.39 |  | 5.14 | 0.64 | *** | 5.69 |
| 95th to <99th | -4.60 | 0.41 | *** | -1.62 | 0.42 | *** | 2.99 | 0.68 | *** | 5.03 |
| ≥99th | -3.91 | 0.36 | *** | -0.36 | 0.37 |  | 3.56 | 0.59 | *** | 4.60 |
| All | 2.25 | 0.23 | *** | 0.75 | 0.22 | *** | -1.50 | 0.39 | *** | 6.76 |
| **BMIp95** | | | | | | | | | | |
| 5th to <85th | 1.61 | 0.08 | *** | -0.63 | 0.08 | *** | -2.24 | 0.13 | *** | 2.56 |
| 85th to <95th | -0.40 | 0.18 |  | 0.23 | 0.18 |  | 0.63 | 0.27 |  | 3.84 |
| 95th to <99th | -0.73 | 0.29 |  | 0.22 | 0.30 |  | 0.96 | 0.44 |  | 4.99 |
| ≥99th | -4.37 | 0.55 | *** | 1.65 | 0.53 | ** | 6.01 | 0.85 | *** | 7.36 |
| All | 0.67 | 0.09 | *** | -0.26 | 0.09 | ** | -0.93 | 0.14 | *** | 3.67 |

** indicates 0.001<p<0.01; *** indicates p<0.001. Only p-values <0.01 are indicated. P-values are obtained from mixed models in which age is modeled using piecewise linear splines. SD, standard deviation.
